# Cadmium stress interacts with nutrient availability and light condition to affect the growth of *Hydrocotyle vulgaris*

**DOI:** 10.1371/journal.pone.0280449

**Published:** 2023-01-18

**Authors:** Rui Zhang, Zhi-Huan Chen, Wen-Tao Cui, Shang-Yan Qiu, Zi-Han Qian, Xue-Ge He, Jun-Cai Xin, Chao Si

**Affiliations:** 1 School of Life Science and Engineering, Handan University, Handan, China; 2 School of Special Education, Handan University, Handan, China; Qinghai University, CHINA

## Abstract

Heavy metal pollution is becoming a serious problem in wetland and often co-occurs with nutrient availability and light conditions variation. We hypothesized that nutrient availability and light condition can affect the growth of wetland plants under heavy metal stress. To test this hypothesis, single ramets of a common, clonal wetland plant *Hydrocotyle vulgaris* were grown for four weeks at three levels of cadmium with three levels of nutrient availability under 30% or 100% light conditions. High level of nutrient availability and high light condition overall promoted growth of *H*. *vulgaris* under Cd stress. Under the two light conditions, responses of *H*. *vulgaris* to Cd treatments differed among three nutrient levels. Under 30% light condition, 2 mg L^-1^ Cd^2+^ treatment decreased total mass at the low nutrient level and decreased ramet number at the medium nutrient level; 0.5 and 2 mg L^-1^ Cd^2+^ treatments decreased leaf mass ratio at the low and the medium nutrient levels. Under 100% light condition, 2 mg L^-1^ Cd^2+^ treatments significantly decreased total mass at the high level of nutrients; 2 mg L^-1^ Cd^2+^ treatment decreased ramet number at the medium and the high nutrient levels and decreased leaf mass ratio at the medium nutrient levels. Our results suggested that Cd stress can interact with nutrient availability and light condition to affect the performance of wetland plants such as *H*. *vulgaris*.

## Introduction

The release of heavy metals into natural habitats from human activities during the process of industrialization and urbanization are increasing [1–3]. Some of heavy metals such as cadmium (Cd), chromium (Cr), lead (Pb) and mercury (Hg) are toxic and nonessential element for plant [3–5]. When plants grow in the environment containing a high level of heavy metal concentration, they may easily absorb such heavy metals and accumulate in the body [6–8]. Excessive heavy metals may adversely affect physiological and biochemical activities in plants and thus inhibiting plant growth and even lead to death [9, 10]. Wetland is one of ecosystems with many important environmental functions and habitats for various plants on the earth [11–14], but is also one of the ecosystems most severely influenced by heavy metals [15, 16]. Heavy metals are discharged into wetland along with agricultural wastewater and industrial sewage, and seriously affect growth and community successions of wetland plants [17–19].

Another seriously environmental problem caused by human activities is the discharge of large amounts of nutrients, such as nitrogen (N), phosphorus (P) and potassium (K) into wetlands, which can lead to eutrophication [6, 20, 21]. Nutrient availability is an important factor for plants which can significantly affect the growth, and may further alter the responses of wetland plants to heavy metals [22–24]. Increasing nutrients within a certain range can alleviate the adverse effects of heavy metals on plants [6, 25–27]. The appropriate added nutrients attenuated the reduction in relative growth rate of *Pistia stratiotes* due to Cr exposure [28]. On the other hand, the excessive nutrient concentration may have toxic effects, and may exacerbate the negative effects of heavy metals on plants [28–30]. Thus, more studies are needed to clarify the effects of nutrient availability on responses of wetland plants to heavy metals.

In addition, shading is a common factor affecting the dwarf herbaceous plants in wetland ecosystems, as they often co-exist with tall emergent plants and the light may shade by canopy of those tall plants [27, 31, 32]. Previous studies have indicated that limited light could reduce the uptake of nutrients by plants, and alter plant response to increased nutrient availability [33–35]. For instance, the responses of biomass, ramet number and mean stolon internode length of *Salvinia natans* to nutrient availability were unimodal under the high light condition, but bimodal under the low light condition [26]. And the response of biomass allocation to nutrients also showed a significant difference between the two light conditions [26]. Based on the above, it is logical to speculate that light and nutrients can both affect plant responses to heavy metal, which however seems to have been ignored before.

A large percentage of wetland plants can clonal growth [14, 36, 37]. They are usually the dominant species in communities, and play an important role in wetlands such as purify the water quality by absorb unnecessary nitrogen, phosphorus etc., and increase dissolved oxygen in the water by release oxygen through the root system [38–40]. And the responses of clonal plants to environmental variation and stresses can further profoundly influence composition and structure of communities in wetland ecosystems [41–43]. Therefore, we need to evaluate the responses of growth, vegetive propagation and biomass allocation in clonal plants to multiple mixed changes of environmental factors in order to make better utilization of these species for phytoremediation and vegetation restoration in polluted wetlands.

In this study, we conducted an experiment in greenhouse to examine the effects of cadmium stress, nutrient availability and light condition on a wetland clonal plant *Hydrocotyle vulgaris* which is common in South China. Ramets of *H*. *vulgaris* were subjected to three levels of Cd concentration crossed with three levels of nutrients availability under low or high light conditions. Specifically, we examined the following hypotheses: (1) increased cadmium concentration can inhibit growth of *H*. *vulgaris*; (2) high light condition can promote performance of *H*. *vulgaris*; (3) *H*. *vulgaris* can grow better under high nutrient level; (4) high nutrient availability and light condition can alleviate inhibition of cadmium stress on *H*. *vulgaris*.

## Materials and methods

### Plant species

*Hydrocotyle vulgaris* L. (Araliaceae) is a perennial clonal herb native to Europe, Southern North America and Central America [26, 27]. The species was introduced to China as an ornamental in the 1990s and was widely distributed in wetlands of regions south of Yangtze River [27]. With the adaptability and phenotypic plasticity, *H*. *vulgaris* can adapt well to a variety of habitats, from terrestrial, and moist to aquatic [26, 27]. Along plagiotropic stolon that can grow either under-ground or aboveground, each node can produce a ramet which only has a petiolate leaf and adventitious roots [28, 29]. The plants of *H*. *vulgaris* we used in the experiment were purchased from a commercial supplier in Zhangzhou, Fujian Province, China. They were cultivated for several weeks in the greenhouse (36° 34′ N, 114° 29′ E) at Handan University in Handan, Hebei Province, China, before the commencement of the experiment.

### Experimental design

The experiment crossed three Cd treatments (non-Cd, 0.5 and 2 mg L^-1^ Cd^2+^) with three nutrient treatments (low, medium and high) and two light treatments (low and high). 90 ramets that had one leaf (the leaf diameter was about 3.8 cm; the petiole was about 15.8 cm) and several adventitious roots were selected and randomly assigned to treatment combinations. Each ramet was placed in a colonization basket which was fixed on a floating plate in the container, thus adventitious roots were kept submerged.

For the three nutrient treatments, respectively, plastic containers (11.8 cm in diameter, 7.6 in deep) was filled with 0 mL (low nutrient level), 2 mL (medium nutrient level) or 10 mL (high nutrient level) of the liquid fertilizer (Miracle-Gro, The Scotts Miracle-Gro Company, USA: N ≥ 4.5 g L^-1^, P_2_O_5_ ≥ 1.0 g L^-1^, K_2_O ≥ 9.5g L^-1^, Mg ≥ 0.5g L^-1^, Fe ≥ 0.1g L^-1^). For the Cd treatments, CdS_2_O_4_·8H_2_O was added to the nutrient solution, giving Cd ion concentrations of 0.5 mg L^-1^ or 2 mg L^-1^. We chose these nutrient and Cd concentrations based on the levels of total nitrogen and Cd allowed in municipal effluents and the levels that constitute pollution in the Environmental Quality Standards for Surface Water in China (http://english.mee.gov.cn/SOE/soechina1997/water/standard.htm). These containers were either placed under 30% ambient light (low light condition) with shade cloth inside the greenhouse or placed under 100% natural light condition (high light condition) inside the greenhouse. Therefore, there were 18 treatments in total. Each treatment was replicated five times, making a total of 90 containers.

This experiment was conducted in the same greenhouse in which the plants were initially cultivated. It started on 30 May and ended on 27 June 2021 and lasted for four weeks. All containers were randomly placed on a bench in the greenhouse. During the experiment, the mean air temperature in the greenhouse was 27.4°C and the mean air humidity was 45.5%. The nutrient solutions were replaced every seven days. Between two operations of nutrient solutions replacements, tap water was added to compensate for the loss due to evapotranspiration and absorption.

### Measurements and data analysis

We harvested the experiment when plants covered the whole water surface in many containers. At harvest, we counted the number of ramets and measured total stolon length of *H*. *vulgaris* in each container. The plants were separated into leaves (the leaves in this experiment include petioles), stolons and roots and then dried at 70°C to a constant weight and weighed. We also calculated leaf mass ratio (leaf dry mas / total dry mass), stolon mass ratio (stolon dry mass / total dry mass) and root mass ratio (root dry mass / total dry mass).

We used a three-way ANOVA to test the effects of Cd treatment, nutrient level, light intensity and their interactions on total mass, leaf mass, stolon mass, root mass, ramet number, total stolon length, leaf mass ratio, stolon mass ratio and root mass ratio of *H*. *vulgaris*. We then used the simple effect test (LSD) to compare the means of each variable among the three levels of Cd concentration within each of the three nutrient levels under the two light conditions. Before analysis, all the data were checked for homoscedasticity and transformed as needed to improve the normality and homoscedasticity of variance: total mass, leaf mass, stolon mass, root mass, ramet number and total stolon length were transformed to the natural log. All the analyses were conducted using SPSS 22.0 (IBM, Inc., Armonk, NY, USA).

## Results

### Effects of Cd, nutrient and light on biomass

**Fig 1:**
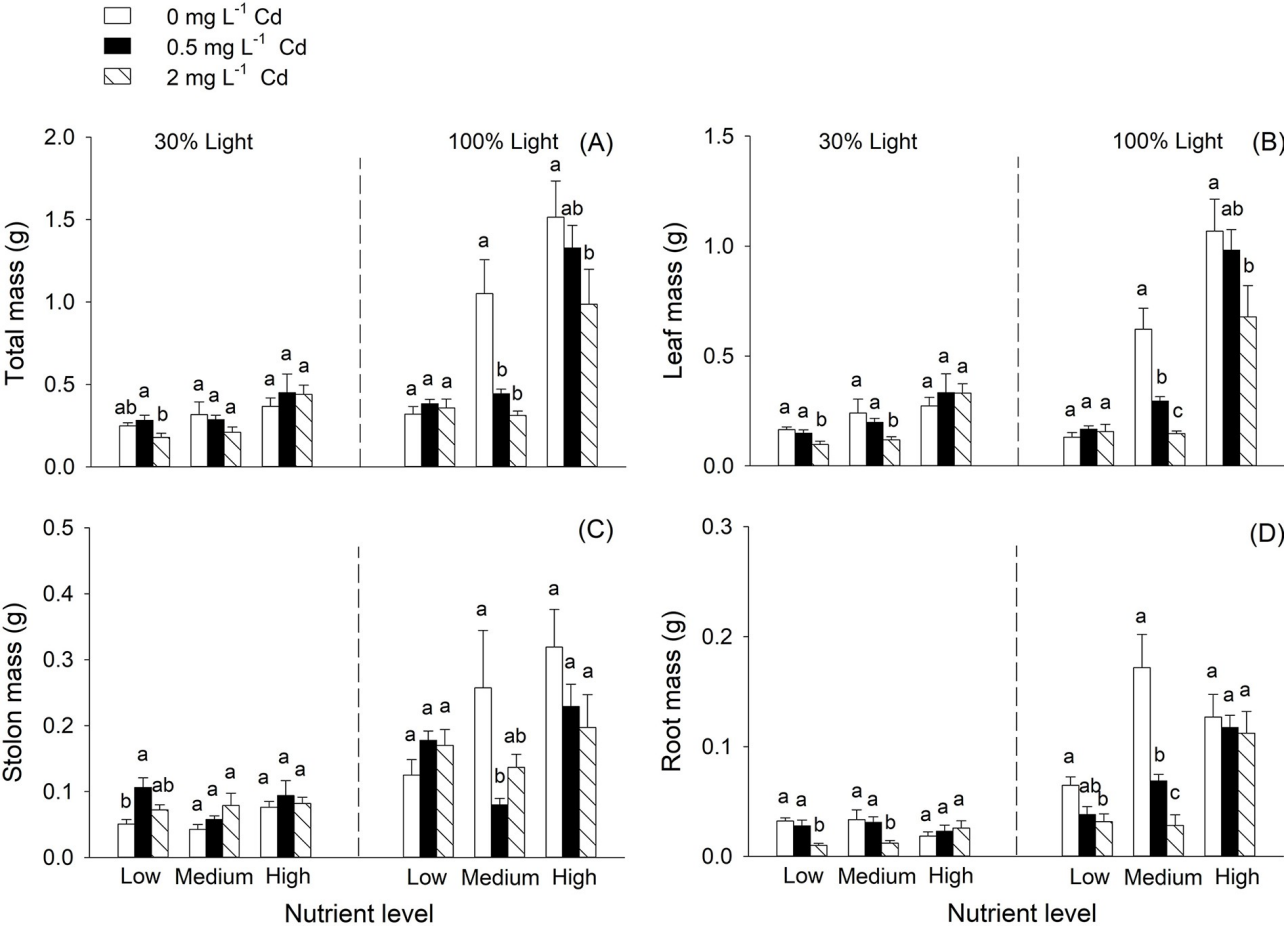
Effects of cadmium concentration, nutrient availability, and light condition on total mass (A), leaf mass (B), stolon mass (C) and root mass (D) of *Hydrocotyle vulgaris*. Bars and vertical lines are means and SE (n = 5). Different small letters (a-c) above bars indicate significant differences among the three levels of Cd concentration within each of the three nutrient levels under the two light conditions.

Cd treatments significantly affected total mass, leaf mass and root mass of *H*. *vulgaris* (Table 1). Nutrient availability and light conditions significantly affected biomass (total mass, leaf mass, stolon mass and root mass, Table 1). In general, the high Cd level decreased total mass, leaf mass and root mass of *H*. *vulgaris* (Table 1, Fig 1A, 1B, 1D). The increased nutrient levels increased biomass of *H*. *vulgaris* (Table 1, Fig 1). *H*. *vulgaris* grew better and produced more biomass under 100% light condition than that under 30% light condition (Table 1, Fig 1). Responses of biomass of *H*. *vulgaris* to Cd treatments varied among different nutrient levels (Fig 1). The interaction between Cd treatments and light conditions significantly affected stolon mass (Table 1). Under 30% light condition, *H*. *vulgaris* produced more stolon mass under 0.5 mg L^-1^ Cd^2+^ treatment at low nutrient level. Under 100% light, *H*. *vulgaris* produced less stolon mass under 0.5 mg L^-1^ Cd^2+^ treatment at medium nutrient level. Nutrient availability and light had a significantly interactive effect on total mass, leaf mass and root mass (Table 1). Three-way interactive effects among Cd treatment, nutrient availability and light condition significantly affected total mass and leaf mass (Table 1). Specifically, under 30% light condition, 2mg L^-1^ Cd treatment significantly decreased total mass at the low nutrient level, whereas under 100% light condition, 2 mg L^-1^ Cd treatment significantly decreased total mass at the medium and high levels of nutrient (Fig 1A). Under 30% light condition, leaf mass was significantly decreased by 2 mg L^-1^ Cd treatment when nutrient levels were low and medium, whereas under 100% light condition, it was significantly decreased by 2 mg L^-1^ Cd treatment at medium and high nutrient levels.

**Table 1:** Effects of cadmium concentration, nutrient availability and light condition on biomass, ramet number, total stolon length and biomass allocation of *Hydrocotyle vulgaris*.

Variable	Cadmium (Cd)		Nutrient (N)		Light (L)		Cd×N		Cd×L		N×L		Cd×N×L		
	F_2, 72_	*P*	F_2, 72_	*P*	F_1, 72_	*P*	F_4, 72_	*P*	F_2, 72_	*P*	F_2, 72_	*P*	F_4, 72_	*P*
Total mass ^a^	**8.7**	**<0.001**	**58.3**	**<0.001**	**114.4**	**<0.001**	**3.4**	**0.013**	2.5	0.092	**8.9**	**<0.001**	**3.1**	**0.020**	
Leaf mass ^a^	**15.3**	**<0.001**	**111.1**	**<0.001**	**64.8**	**<0.001**	**5.1**	**0.001**	1.2	0.320	**16.0**	**<0.001**	**4.1**	**0.005**	
Stolon mass ^a^	0.3	0.753	**9.0**	**<0.001**	**79.2**	**<0.001**	**2.7**	**0.036**	**4.0**	**0.023**	0.9	0.420	0.9	0.471	
Root mass ^a^	**23.0**	**<0.001**	**8.8**	**<0.001**	**142.2**	**<0.001**	**8.0**	**<0.001**	2.3	0.103	**10.2**	**<0.001**	1.9	0.125	
Ramet number ^a^	**12.3**	**<0.001**	**81.6**	**<0.001**	**98.5**	**<0.001**	**5.4**	**0.001**	0.3	0.730	**14.2**	**<0.001**	**4.2**	**0.004**	
Total stolon length ^a^	**32.2**	**<0.001**	**84.2**	**<0.001**	**98.9**	**<0.001**	**5.0**	**0.001**	2.3	0.106	**9.7**	**<0.001**	2.2	0.073	
Leaf mass ratio	**20.0**	**<0.001**	**184.3**	**<0.001**	**90.4**	**<0.001**	**10.0**	**<0.001**	**9.9**	**<0.001**	**13.5**	**<0.001**	**2.8**	**0.033**	
Stolon mass ratio	**41.0**	**<0.001**	**106.9**	**<0.001**	**20.3**	**<0.001**	**20.7**	**<0.001**	**4.1**	**0.021**	**11.8**	**<0.001**	1.5	0.197	
Root mass ratio	**13.2**	**<0.001**	**14.8**	**<0.001**	**39.4**	**<0.001**	**9.5**	**<0.001**	1.6	0.201	<0.1	0.968	1.4	0.236	

^a^ Natural log transformation. F, *P* values and degree of freedom (subscript for “F”) are provided. Values are in bold when *P* < 0.05.

### Effects of Cd, nutrient and light on ramet number and total stolon length

**Fig 2:**
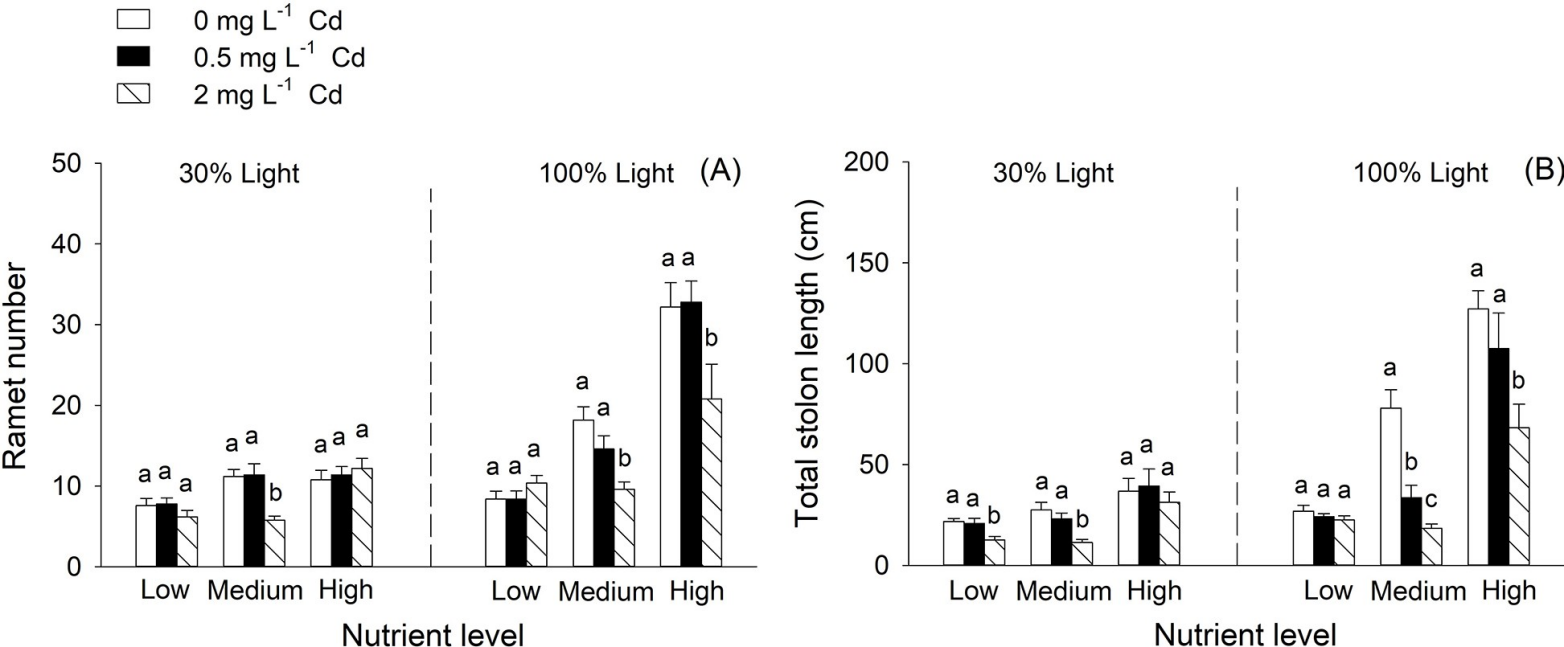
Effects of cadmium concentration, nutrient availability and light condition on ramet number (A) and total stolon length (B) of *Hydrocotyle vulgaris*. Bars and vertical lines are means and SE (n = 5). Different small letters (a-c) above bars indicate significant differences among the three levels of Cd concentration within each of the three nutrient levels under the two light conditions.

Under 100% light condition, *H*. *vulgaris* produced more ramets and total stolon length than that under 30% light condition (Table 1, Fig 2). Both ramet number and total stolon length were significantly decreased at the high Cd level (Table 1, Fig 2), whereas this effect was alleviated by increasing nutrient availability (Table 1, Fig 2). Responses of ramet number and total stolon length to Cd treatment varied among different nutrient levels (Table 1, Fig 2). Nutrient availability and light condition had significantly interactive effects on ramet number and total stolon length (Table 1, Fig 2). Three-way interactive effect among Cd treatment, nutrient availability and light condition significantly affected ramet number (Table 1). Under 30% light condition, ramet number was significantly decreased by 2 mg L^-1^ Cd treatment at medium nutrient level, whereas under 100% light condition, it was decreased by 2mg L^-1^ Cd treatment at medium and high nutrient levels (Fig 2A).

### Effects of Cd, nutrient and light on biomass allocation

**Fig 3:**
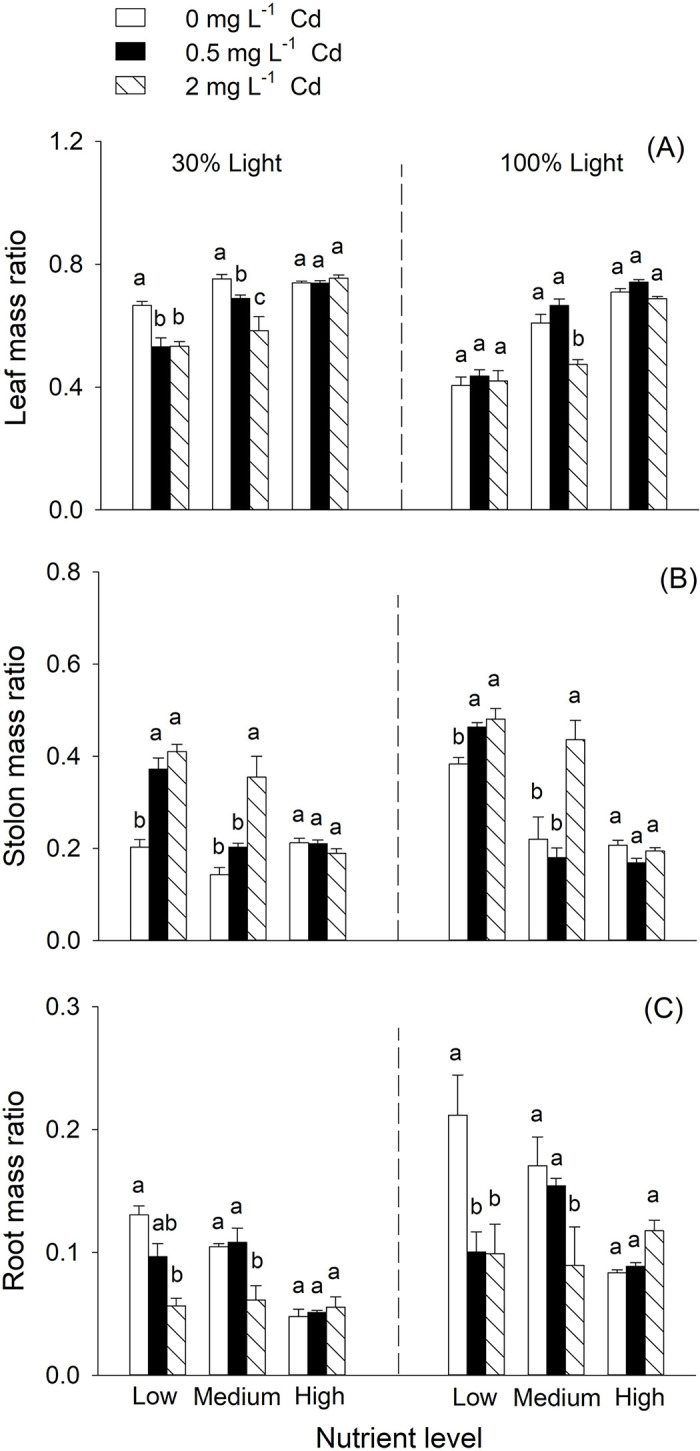
Effects of cadmium concentration, nutrient availability and light condition on leaf mass ratio (A), stolon mass ratio (B), and root mass ratio (C) of *Hydrocotyle vulgaris*. Bars and vertical lines are means and SE (n = 5). Different small letters (a-c) above bars indicate significant differences among the three levels of Cd concentration within each of the three nutrient levels under the two light conditions.

*H*. *vulgaris* allocated more biomass to stolon and root and less biomass to leaf under 100% light condition than that under 30% light condition (Table 1, Fig 3). Overall, 2 mg L^-1^ Cd treatment decreased leaf mass ratio and root mass ratio, but increased stolon mass ratio of *H*. *vulgaris* (Fig 3). Increased nutrient availability tended to increase leaf mass ratio but tended to decrease stolon mass ratio and root mass ratio (Fig 3). Responses of biomass allocation to Cd treatments significantly differed among three nutrient levels (Table 1, Fig 3). Both two-way interactive effects between Cd treatment and light condition and two-way interactive effects between nutrient availability and light condition significantly affected leaf mass ratio and stolon mass ratio (Table 1). Three-way interaction effects among Cd treatment, nutrient availability and light condition significantly affected leaf mass ratio (Table 1). Under 30% light condition, leaf mass ratio tended to be decreased with 0.5 and 2 mg L^-1^ Cd^2+^ treatment at low and medium nutrient levels, whereas under 100% light condition, it tended to be decreased with 2 mg L^-1^ Cd^2+^ treatment at medium nutrient level (Fig 3A).

## Discussion

Increased Cd level significantly inhibited growth of *H*. *vulgaris*, which is consistent with some studies on other wetland plant species [6, 44, 45]. For instance, *Typha latifolia* showed significantly negative responses to the high level of Cd concentration (30 mg/kg Cd in wetland soil) in terms of root and shoot lengths and mass [46]. The shoot and root mass under the high Cd level was only 13.2–30.9% of that under the treatment without Cd [46]. Cd is a non-essential element in plants [32, 33]. The adverse effects of Cd on *H*. *vulgaris* is likely to be that the excessive Cd is toxic to plants due to its influence on various physiological activities such as inhibiting chlorophyll synthesis and photosynthesis, then altering the activity of many key enzymes, hampering nutrient uptake and secondary metabolism and then inhibiting plant growth [46–48].

As predicted, high nutrient availability promoted growth of *H*. *vulgaris* under Cd stress. Similar results have been shown in previous studies [6]. Adomako et al. reported that in Cd polluted water *Pistia stratiotes* produced 70% - 100% more biomass when a higher level of nutrient was provided [6]. In another study, regardless of the addition of nitrogen and phosphorus fertilizer alone or combined nitrogen and phosphorus fertilization, the biomass of plants under heavy metal (Cd, Cu and Pb) treatment was increased [28, 49]. The reason for the positive effect of high nutrient availability is perhaps that it can dilute Cd concentration in plants and mitigate the toxicity of Cd to plants by promoting plant growth and reducing uptake and accumulation of Cd in plants [50–54].

High light condition similarly increased growth of *H*. *vulgaris* under Cd stress. High light perhaps enhanced photosynthesis efficiency and then promoted the growth of *H*. *vulgaris* to alleviate the negative effect of Cd stress [55–57]. The low light intensity caused by shading frequently occurs in wetlands [58, 59]. Interactions of light condition and heavy metals have been well studied on phytoplankton such as *Synechocystis* sp. and *Euglena gracilis* [60, 61], but little has been studied on herbaceous plant. Our result suggested that light is an important factor affecting plant response to heavy metals and should be considered when such plants are used to restore the heavy metal polluted wetlands.

*H*. *vulgaris* produced more total mass at the high level of nutrient availability and light condition than that at other levels of nutrients and light. There were no significant differences among the three Cd treatments when levels of nutrient availability and light condition were both high. In addition, under the two light conditions, responses of growth to Cd treatments varied among different nutrient levels. For instance, under low light condition, high levels of Cd showed a negative effect on total mass at a low level of nutrient availability, whereas under high light condition, high levels of Cd significantly reduced total mass at medium and high levels of nutrient availability. These results suggested that the impact of Cd stress on plant growth could vary with nutrient availability and light condition. However, we do not know the exact mechanism underlying the three-way interactive effects of Cd stress, nutrient availability and light condition.

Overall, increased nutrients and high light can alleviate adverse effects of Cd stress on growth of *H*. *vulgaris* and may improve the adaptability and tolerance of plants to Cd exposure [49]. Therefore, one potential application is that sufficient nutrients and light can increase the efficiency of plants to restore the polluted wetland by accumulating heavy metals [6, 23]. In addition, the increased nutrients and high light may significantly facilitate the expansion of clonal plants in heavy metal polluted wetlands, which can further affect the interspecific competition and plant community dynamics [45, 62].

We also found that Cd stress interacted with nutrient availability and light condition to affect leaf mass ratio of *H*. *vulgaris*. Biomass allocation is one of the adaptive strategies for balancing plant growth and resource restriction [62–64]. In this study, *H*. *vulgaris* allocate more biomass to roots but less to leaves under low light condition. A similar response of biomass allocation was found in another wetland plant *Salvinia natans* [26]. These results could be explained by the typical resource-ratio hypothesis, i.e., plants tend to allocate more biomass to aboveground organs to compete for light when light is limited [65]. Under different light conditions, responses of leaf mass ratio to Cd treatment were different at different nutrient levels. The results suggested that wetland plants could alter their biomass allocation to adapt to environmental stress or variation [56, 66, 67]. The underlying mechanism is unclear and deserves further studies.

## Conclusion

We conclude that Cd stress can interact with nutrient availability and light condition to affect the performance of wetland plants. Sufficient nutrient availability and light condition can alleviate the adverse effect of Cd stress on wetland plants. However, we still lack the specific mechanistic understanding of such combined effects. Further studies were needed to examine such underlying mechanisms to give insights into the impact of Cd stress on performance of wetland plants.
